# Challenges and Facilitation Approaches for the Participatory Design of AI-Based Clinical Decision Support Systems: Protocol for a Scoping Review

**DOI:** 10.2196/58185

**Published:** 2024-09-05

**Authors:** Tabea Rambach, Patricia Gleim, Sekina Mandelartz, Carolin Heizmann, Christophe Kunze, Philipp Kellmeyer

**Affiliations:** 1 Care & Technology Lab Furtwangen University Furtwangen Germany; 2 Human-Technology Interaction Lab Department of Neurosurgery University Medical Center Freiburg Freiburg im Breisgau Germany; 3 Data and Web Science Group School of Business Informatics and Mathematics University of Mannheim Mannheim Germany

**Keywords:** artificial intelligence, AI, participation, participatory design, co-creation, clinical decision support system, CDSS, decision support, challenges, clinical staff, scoping review

## Abstract

**Background:**

In the last few years, there has been an increasing interest in the development of artificial intelligence (AI)–based clinical decision support systems (CDSS). However, there are barriers to the successful implementation of such systems in practice, including the lack of acceptance of these systems. Participatory approaches aim to involve future users in designing applications such as CDSS to be more acceptable, feasible, and fundamentally more relevant for practice. The development of technologies based on AI, however, challenges the process of user involvement and related methods.

**Objective:**

The aim of this review is to summarize and present the main approaches, methods, practices, and specific challenges for participatory research and development of AI-based decision support systems involving clinicians.

**Methods:**

This scoping review will follow the Joanna Briggs Institute approach to scoping reviews. The search for eligible studies was conducted in the databases MEDLINE via PubMed; ACM Digital Library; Cumulative Index to Nursing and Allied Health; and PsycInfo. The following search filters, adapted to each database, were used: Period January 01, 2012, to October 31, 2023, English and German studies only, abstract available. The scoping review will include studies that involve the development, piloting, implementation, and evaluation of AI-based CDSS (hybrid and data-driven AI approaches). Clinical staff must be involved in a participatory manner. Data retrieval will be accompanied by a manual gray literature search. Potential publications will then be exported into reference management software, and duplicates will be removed. Afterward, the obtained set of papers will be transferred into a systematic review management tool. All publications will be screened, extracted, and analyzed: title and abstract screening will be carried out by 2 independent reviewers. Disagreements will be resolved by involving a third reviewer. Data will be extracted using a data extraction tool prepared for the study.

**Results:**

This scoping review protocol was registered on March 11, 2023, at the Open Science Framework. The full-text screening had already started at that time. Of the 3,118 studies screened by title and abstract, 31 were included in the full-text screening. Data collection and analysis as well as manuscript preparation are planned for the second and third quarter of 2024. The manuscript should be submitted towards the end of 2024.

**Conclusions:**

This review will describe the current state of knowledge on participatory development of AI-based decision support systems. The aim is to identify knowledge gaps and provide research impetus. It also aims to provide relevant information for policy makers and practitioners.

**International Registered Report Identifier (IRRID):**

DERR1-10.2196/58185

## Introduction

### Background

Clinical decision support systems (CDSS) play an important role in health care by providing evidence-based guidance and recommendations to clinical staff. Typical use cases include medication management, disease diagnosis and management, treatment planning, risk assessment, and workflow optimization. The use of these systems aims to increase the accuracy and effectiveness of clinical staff [1,2].

The increasing digital transformation of health care means that more and more health care data is available in digital form, which means that artificial intelligence (AI) applications are also becoming more relevant in this area. More and more AI applications are being developed in health care. In clinical practice, AI has a wide range of potential applications, including disease diagnosis, treatment selection, and patient monitoring [3]. AI-based CDSS is essential in this context.

Although studies have shown that CDSS can reduce medical errors and improve outcomes, they have also shown that CDSS are not being used to their full potential [4-10]. It can be assumed that the challenges of non–AI-based CDSS also apply to AI-based CDSS. Further challenges arise with respect to AI-based CDSS [11]: pointed out in the viewpoint is that the use of deep learning and other analytic methods brings additional challenges. These methods generate insights in ways that are not directly traceable, meaning that clinical staff cannot apply the same validation as with traditional clinical decision support tools. As a result of this lack of transparency, trust in the AI system may decrease [11,12].

Another challenge is integration into workflows [13,14]. When developing AI-based technologies for clinical use, it is crucial to consider existing workflows in both the design and development phases. This will ensure that the technology can be used effectively and make a positive contribution to patient care. Consideration of workflows and the needs of clinical staff will ensure the successful integration of AI technologies into everyday clinical practice. Poor integration processes can have a negative impact on uptake and adoption, as illustrated by a case study on the implementation of a CDSS [14]. For successful integration, it is essential that clinical staff develop the skills to interpret the results appropriately. This “black box” nature of AI described by [11] and the lack of transparency of the basis for decision making can make practical implementation difficult and may also be a factor in low user adoption [11,15].

The notion of the user’s acceptance of new technologies is derived from the Theory of Reasoned Action [16]. According to various technology acceptance models (TAMs; eg, TAM and UTAUT [Unified Theory of Acceptance and Use of Technology]) based on this theory, acceptance encompasses both the intention to use technology and its influence on actual use behavior [4,17,18]. For researching clinicians’ acceptance of CDSS these models serve as a foundation [19-22].

Acceptance of CDSSs is crucial for their successful use. Involving users at an early stage can improve the acceptance and use of information technology by taking into account their needs, preferences, and experiences. This can help to optimize the user experience and increase the effectiveness of the technologies [15,17].

Involving future users is possible through participatory research approaches. Such approaches aim to plan and carry out research processes with people who investigate their social world and meaningful actions as lifeworld-situated living and working practices that are to be improved by developing appropriate innovations [23]. In addition, a participatory approach may also help to avoid problems that arise when transferring the AI-based CDSS to new patient populations for instance due to overfitting of training data or lack of generalizability [24]. This problem can be counteracted by involving expert knowledge from practice, for instance by reviewing the operationalization of health care–related concepts, feature selection, and data quality.

The stage of participation can be determined using the Wright et al [25] stage model. This model consists of 9 stages and is divided into four areas: (1) nonparticipation (stages 12), (2) preparticipation (stages 3-5), (3) participation (stages 6-8), and (4) beyond participation (stage 9). It is also used to determine the stage of participation in the studies reviewed. In order to be able to categorize the studies, particular attention should be paid to the methodological description of the studies. The second version of the GRIPP (Guidance for Reporting Involvement of Patients and Public), a tool to improve the reporting of patient and public involvement in research, shows in its fourth section of the long-form reporting checklist on the methodology of the work (design, people involved, stages of involvement, and level or type of involvement) the relevant aspects for describing the involvement of groups of people [26]. The content can also be transferred to other stakeholder groups (not only patient and public involvement). An alternative to the Wright et al [25] stage model and GRIPP 2 could have been the participatory ergonomics framework, which focuses on the active involvement of participants, particularly workers, in ergonomic interventions to improve work conditions and processes [27]. However, the Wright model and GRIPP 2 were selected because of their established use in health care, their broad applicability, their transferability to different stakeholder groups, and their alignment with the objectives of this review.

According to a recent review, clinical professionals (future users) are already involved in the development of AI-based CDSS, but only in about 30% of cases. The focus is on the creation of predictive CDSS specifications or the evaluation of system implementations. However, clinical experts are less likely to be involved in the development phases to check clinical validity, select model features, process data, or act as a gold standard [28].

Nevertheless, the development of AI applications poses challenges to participatory methods: A precondition for carrying out participatory methods on the topic of AI is that the participants have a basic understanding of AI. Furthermore, implementing design ideas is difficult, as a realistic prototype is often hard to realize. In addition, evaluating the results is only possible to a limited extent. Much has to be done via simulations or imagination because it often requires a long testing period. Another difficulty is often the lack of comprehensibility of the AI decisions for the user [29].

Although clinical staff are already involved in the research and technology development process of AI-based CDSS, no overarching overview has been identified that summarizes and presents the main approaches, methods, and practices for participatory research and technology development of AI-based CDSS for clinical staff. Hence, this gap of knowledge should be addressed by this scoping review.

A preliminary search of MEDLINE was conducted, and no current or ongoing systematic reviews or scoping reviews on the topic were identified.

### Objectives and Research Questions

The objective of the review is to provide an overview and systematization of participatory approaches of various disciplines for developing, piloting, implementing, and evaluating AI-based information systems in a clinical setting.

The following research questions will be addressed:

1. Perspectives on the underlying clinical problem: Are the different perspectives on underlying clinical problems (eg, by nurses, doctors, and other health care workers) addressed by any particular CDSS included in the design of the CDSS?
2. Participation as a process: Which participatory approaches are used to develop, pilot, and evaluate AI-based CDSS in health care?
3. Participation in technical aspects of CDSS design: In which ways are participatory methods specifically supporting the development of AI components of CDSS and their performance?
4. Participation for ethical, legal, and social implications: Which ethical, legal, and social implications have been identified in existing projects for participatory development, piloting, implementation, and evaluation processes targeting clinical staff?

## Methods

### Design

We are going to conduct one scoping review. The PRISMA-ScR (Preferred Reporting Items for Systematic Reviews and Meta-Analyses extension for Scoping Reviews) is used as a basic tool [30] in combination with the Joanna Briggs Institute (JBI) approach to scoping reviews. This approach ensures that the scoping review is transparent, reproducible, and methodologically sound [31].

### Search Strategy and Terms

#### Information Sources

To identify relevant papers, we have chosen various databases that encompass publications from the fields of biomedical science and health science, computer science and information technology, psychology, and nursing and allied health. The following electronic databases will be included as information sources: MEDLINE via PubMed, ACM Digital Library, Cumulative Index to Nursing and Allied Health, and PsycInfo. Additionally, we will supplement this research with the snowball technique [32] and the screening of websites (eg, Google Scholar and DAHTA). These information sources were chosen since they encompass a wide range of research fields considered appropriate to address the objectives of this review. The databases provide the most comprehensive coverage of relevant studies examining the participatory design and development of AI-based technologies in health care, particularly CDSS.

#### Search Strategy

The above sources will be searched using combinations of relevant search terms we developed and tested for sensitivity before performing the scoping review. We used an iterative approach to develop the search strategy. First, we identified search terms used in previous studies and reviews related to participatory research and AI-based CDSS for clinical staff (particularly relevant: [33-37]). Then, we conducted an initial search in MEDLINE (via PubMed) and Cumulative Index to Nursing and Allied Health after analyzing text words (title and abstract) and indexed terms, as suggested by the Joanna Briggs Institute methodology for systematic scoping reviews [31,38]. Based on these results, we used the search terms in all databases. Afterward, we will check the references for all included contributions. If relevant, we will contact the authors. We will contact the authors if a publication is inaccessible or further information is required. Other reasons for contacting authors might include clarifying any ambiguous or unclear data presented in their publication, requesting additional data that may not have been included in the original publication, or seeking permission to use specific figures, tables, or other content.

Multimedia Appendix 1 demonstrates the search strategy for MEDLINE (via PubMed). The research began in 2012, the year in which the use of deep neural networks in image processing marked a breakthrough in the field of deep learning [39]. The terms will be adapted to the basic search particulars (eg, wildcards [*]and truncations) of each electronic database.

In order to describe the inclusion criteria precisely, we rely on the Population, Concept, and Context scheme [31,38]. Textbox 1 shows the most important criteria according to the Population, Concept, and Context scheme.

**Population, Concept, and Context criteria used in the scoping review.**

**Population**

Clinical staff (medical doctors, nurses,...)

**Concept**

Participation/participatory design/Co-creation/co-design.

**Context**

Development, piloting, implementation, and evaluation of artificial intelligence–based clinical decision support systems (hybrid and data-driven artificial intelligence approaches).

**Types of sources**

Primary research—All study types (eg, qualitative, quantitative, mixed methods) will be included. Systematic reviews and meta-analyses will be used for manual searches in the reference lists to identify further primary studies.

Papers that provide information on at least 1 research question should be included. More specific inclusion and exclusion criteria are provided below for each review.

### Eligibility Criteria

The inclusion and exclusion criteria that were applied to the studies are shown in Textbox 2.

**Inclusion and exclusion criteria applied in the scoping review.**

**Inclusion criteria**

Target group: Clinical staff.

Involvement: Participation in the development, design, piloting, and evaluation of artificial intelligence–based information systems.

Related approaches: Other related approaches, research and design strategies, or concepts often used interchangeably with participation and co-creation, such as co-design.

Type of research: Primary research using different methods (eg, qualitative, quantitative, and mixed methods)

Language of publications: English or German.

**Exclusion criteria**

Participation: No evidence of a participatory research element.

Target group: No relation to clinical staff.

Thematic focus: Does not refer to artificial intelligence–based clinical decision support systems.

### Study Selection

The retrieved references will be checked for duplicates and transmitted to Covidence (software for managing and streamlining reviews, operated by Veritas Health Innovation Ltd) [40] for the screening steps. We will use Zotero (a free and open-source literature management program) [41] as a bibliographic tool. Two independent reviewers (TR and PG) will screen all titles and abstracts separately for inclusion or exclusion. Disagreements will be solved by including a third reviewer. Afterward, the same procedure will be applied to the full-text screening, which is carried out by 3 independent reviewers (TR, PG, and CH). Reasons for excluding a study will be assessed in each of these steps. The results of the search and the study inclusion process will be reported in full in the final scoping review and presented in a PRISMA-ScR flow diagram [30].

### Data Extraction

A data charting form has been developed jointly by the authors to identify the variables to be extracted. The 2 reviewers will chart the data independently, discuss the results, and continually update the data charting form in an iterative process, with changes detailed in the scoping review. Any reviewer disagreements will be resolved by discussion or with additional reviewers. Where appropriate, authors of papers will be contacted to request missing or additional data as required. A draft extraction form is provided (Multimedia Appendix 2). Data on the participation process is also extracted to assess the stage of participation.

### Data Analysis and Presentation

Data Analysis and presentation will follow the recommendations of the Joanna Briggs Institute scoping review methodology group [42]. First, the extracted data will be presented in a logical and descriptive way (diagrams and tables), guided by the objectives and questions of the scoping review. Additional relevant data items may be identified during the data extraction process. If additional items are extracted that were not prespecified in the review protocol, this will be made clear in the final report together with a rationale as to why it occurred. The extracted data will be summarized in a narrative synthesis to bring together findings relating to challenges and facilitators for participatory design processes. Given the breadth of scoping review questions, the analysis will also use qualitative content analysis. In order to identify and structure relevant aspects of the research questions, the analysis will follow an inductive approach. Following an open coding process, a coding framework will be developed and reviewed by all authors. This approach aims to provide insights into participatory design and research practice for AI-based CDSS and highlight areas for future research.

## Results

This review protocol was submitted to the Open Science Framework on March 11, 2024. The full-text screening had already started at that time. Of the 3,118 studies screened by title and abstract, 31 were included in the full-text screening. Data collection and analysis as well as manuscript preparation are planned for the second and third quarter of 2024. The manuscript should be submitted towards the end of 2024.

## Discussion

### Principal Results

The main objective of this study is to identify, clarify, and map key approaches, methods, and procedures for participatory research and technology development of AI-based CDSS in the context of clinical staff. It will also analyze, synthesize, and develop existing approaches, concepts, and conceptualizations. The insights gained from this process will serve as a basis for designing, developing, and testing participatory processes specifically designed for clinical staff. Various stakeholders can use the results to design, develop, and review participatory processes that address the development of an AI-based CDSS for clinical staff.

### Limitations

Scoping reviews have limitations, particularly in that they focus on the collection and synthesis of data and do not assess the strength of evidence or the risk of bias in the research. Therefore, further research is needed to assess and analyze the quality of existing studies on the participatory development of AI-based decision support systems. It should be noted that only papers written in English or German were considered, which meant that potentially relevant studies in other languages could not be included. Furthermore, despite the comprehensive inclusion criteria, some relevant sources of information may not be included.

### Conclusions

Up to now, no review of this scope and objective has been identified. Hence, this review will be the first to address this specific knowledge gap targeting clinical staff. Additionally, one aim of this review is to identify further research and knowledge gaps and to give hints where further reviews would be helpful.

## Data Availability

All data collected and analyzed during our scoping review will be available on the Open Science Framework repository and included as Multimedia Appendix files with our scoping review publication.

## Appendix

Multimedia Appendix 1 Search strategy.

Multimedia Appendix 2 Data extraction form.

## Abbreviations

AI: artificial intelligence
CDSS: clinical decision support system
GRIPP: Guidance for Reporting Involvement of Patients and Public
JBI: Joanna Briggs Institute
PRISMA-ScR: referred Reporting Items for Systematic Reviews and Meta-Analyses extension for Scoping Reviews
TAM: technology acceptance model
UTAUT: Unified Theory of Acceptance and Use of Technology

## References

[1] Teufel A, Binder H (2021). Clinical decision support systems. Visc Med.

[2] Giger ML (2018). Machine learning in medical imaging. J Am Coll Radiol.

[3] Yu KH, Beam AL, Kohane IS (2018). Artificial intelligence in healthcare. Nat Biomed Eng.

[4] Davis FD (1993). User acceptance of information technology: system characteristics, user perceptions and behavioral impacts. Int J Man-Mach Stud.

[5] Kaushal R, Shojania KG, Bates DW (2003). Effects of computerized physician order entry and clinical decision support systems on medication safety: a systematic review. Arch Intern Med.

[6] Kawamoto K, Houlihan CA, Balas EA, Lobach DF (2005). Improving clinical practice using clinical decision support systems: a systematic review of trials to identify features critical to success. BMJ.

[7] Jaspers MWM, Smeulers M, Vermeulen H, Peute LW (2011). Effects of clinical decision-support systems on practitioner performance and patient outcomes: a synthesis of high-quality systematic review findings. J Am Med Inform Assoc.

[8] Eberhardt J, Bilchik A, Stojadinovic A (2012). Clinical decision support systems: potential with pitfalls. J Surg Oncol.

[9] Castaneda C, Nalley K, Mannion C, Bhattacharyya P, Blake P, Pecora A, Goy A, Suh KS (2015). Clinical decision support systems for improving diagnostic accuracy and achieving precision medicine. J Clin Bioinforma.

[10] Belard A, Buchman T, Forsberg J, Potter BK, Dente CJ, Kirk A, Elster E (2017). Precision diagnosis: a view of the clinical decision support systems (CDSS) landscape through the lens of critical care. J Clin Monit Comput.

[11] Maddox TM, Rumsfeld JS, Payne PRO (2019). Questions for artificial intelligence in health care. JAMA.

[12] Amann J, Vayena E, Ormond KE, Frey D, Madai VI, Blasimme A (2023). Expectations and attitudes towards medical artificial intelligence: a qualitative study in the field of stroke. PLoS One.

[13] Cai CJ, Winter S, Steiner D, Wilcox L, Terry M (2019). "Hello AI": uncovering the onboarding needs of medical practitioners for human-AI collaborative decision-making. Proc ACM Hum-Comput Interact.

[14] Salwei ME, Carayon P (2022). A sociotechnical systems framework for the application of artificial intelligence in health care delivery. J Cogn Eng Decis Mak.

[15] Khairat S, Marc D, Crosby W, Al Sanousi A (2018). Reasons for physicians not adopting clinical decision support systems: critical analysis. JMIR Med Inform.

[16] Fishbein M, Ajzen I (1980). Belief, Attitude, Intention, and Behavior: An Introduction to Theory and Research.

[17] Venkatesh V, Morris MG, Davis GB, Davis FD (2003). User acceptance of information technology: toward a unified view. MIS Q.

[18] Davis FD (1989). Perceived usefulness, perceived ease of use, and user acceptance of information technology. MIS Q.

[19] Jansen-Kosterink S, van Velsen L, Cabrita M (2021). Clinician acceptance of complex clinical decision support systems for treatment allocation of patients with chronic low back pain. BMC Med Inform Decis Mak.

[20] Arts DL, Medlock SK, van Weert HCPM, Wyatt JC, Abu-Hanna A (2018). Acceptance and barriers pertaining to a general practice decision support system for multiple clinical conditions: a mixed methods evaluation. PLoS One.

[21] Heselmans A, Aertgeerts B, Donceel P, Geens S, Van de Velde S, Ramaekers D (2012). Family physicians' perceptions and use of electronic clinical decision support during the first year of implementation. J Med Syst.

[22] Peleg M, Shachak A, Wang D, Karnieli E (2009). Using multi-perspective methodologies to study users' interactions with the prototype front end of a guideline-based decision support system for diabetic foot care. Int J Med Inform.

[23] Bergold J, Thomas S (2012). Participatory research methods: a methodological approach in motion. JSTOR.

[24] Moazemi S, Vahdati S, Li J, Kalkhoff S, Castano LJV, Dewitz B, Bibo R, Sabouniaghdam P, Tootooni MS, Bundschuh RA, Lichtenberg A, Aubin H, Schmid F (2023). Artificial intelligence for clinical decision support for monitoring patients in cardiovascular ICUs: a systematic review. Front Med (Lausanne).

[25] Wright M, Block M, von Unger H (2008). Participation in the cooperation between target group, project and sponsor. Gesundheitswesen.

[26] Staniszewska S, Brett J, Simera I, Seers K, Mockford C, Goodlad S, Altman DG, Moher D, Barber R, Denegri S, Entwistle A, Littlejohns P, Morris C, Suleman R, Thomas V, Tysall C (2017). GRIPP2 reporting checklists: tools to improve reporting of patient and public involvement in research. BMJ.

[27] Haines H, Wilson JR, Vink P, Koningsveld E (2002). Validating a framework for participatory ergonomics (the PEF). Ergonomics.

[28] Schwartz JM, Moy AJ, Rossetti SC, Elhadad N, Cato KD (2021). Clinician involvement in research on machine learning-based predictive clinical decision support for the hospital setting: a scoping review. J Am Med Inform Assoc.

[29] Bratteteig T, Verne G, Huybrechts L, Teli M, Light A, Lee Y, Di Salvo C, Grönvall E, Kanstrup AM, Bødker K (2018). Does AI make PD obsolete?. Proceedings of the 15th Participatory Design Conference: Short Papers, Situated Actions, Workshops and Tutorial - Volume 2.

[30] Tricco AC, Lillie E, Zarin W, O'Brien KK, Colquhoun H, Levac D, Moher D, Peters MDJ, Horsley T, Weeks L, Hempel S, Akl EA, Chang C, McGowan J, Stewart L, Hartling L, Aldcroft A, Wilson MG, Garritty C, Lewin S, Godfrey CM, Macdonald MT, Langlois EV, Soares-Weiser K, Moriarty J, Clifford T, Tunçalp Ö, Straus SE (2018). PRISMA extension for scoping reviews (PRISMA-ScR): checklist and explanation. Ann Intern Med.

[31] Peters MDJ, Godfrey CM, McInerney P, Soares CB, Khalil H, Parker D (2015). Methodology for JBI scoping reviews. The Joanna Briggs Institute Reviewers’ Manual.

[32] Greenhalgh T, Potts HWW, Wong G, Bark P, Swinglehurst D (2009). Tensions and paradoxes in electronic patient record research: a systematic literature review using the meta-narrative method. Milbank Q.

[33] Ballard S, Chappell K, Kennedy K, Harrison S, Bardzell S, Neustaedter C, Tatar D (2019). Judgment call the game. Proceedings of the 2019 on Designing Interactive Systems Conference.

[34] Kocaballi AB, Ijaz K, Laranjo L, Quiroz JC, Rezazadegan D, Tong HL, Willcock S, Berkovsky S, Coiera E (2020). Envisioning an artificial intelligence documentation assistant for future primary care consultations: a co-design study with general practitioners. J Am Med Inform Assoc.

[35] Clar C, Wright MT (2020). Partizipative Forschung im deutschsprachigen Raum - eine Bestandsaufnahme.

[36] Kasberg A, Müller P, Markert C, Bär G (2021). Categorizing methods used in participatory research [Systematisierung von Methoden partizipativer Forschung]. Bundesgesundheitsblatt Gesundheitsforschung Gesundheitsschutz.

[37] Moore G, Wilding H, Gray K, Castle D (2019). Participatory methods to engage health service users in the development of electronic health resources: systematic review. J Particip Med.

[38] Peters MDJ, Godfrey CM, Khalil H, McInerney P, Parker D, Soares CB (2015). Guidance for conducting systematic scoping reviews. Int J Evid Based Healthc.

[39] Krizhevsky A, Sutskever I, Hinton G, Pereira F, Burges CJ, Bottou L, Weinberger KQ (2012). ImageNet classification with deep convolutional neural networks. Advances in Neural Information Processing Systems.

[40] (2014). Covidence Systematic Review Software.

[41] (2023). Zotero.

[42] Pollock D, Peters MDJ, Khalil H, McInerney P, Alexander L, Tricco AC, Evans C, Godfrey CM, Pieper D, Saran A, Stern C, Munn Z, de Moraes (2023). Recommendations for the extraction, analysis, and presentation of results in scoping reviews. JBI Evid Synth.

