# Supplementary material for: Challenges and Facilitation Approaches for the Participatory Design of AI-Based Clinical Decision Support Systems: Protocol for a Scoping Review
Source: JMIR Res Protoc. 2024 Sep 5;13:e58185. doi: 10.2196/58185 (PMC11413541; doi:10.2196/58185)
Supplement: Multimedia Appendix 1 [file resprot_v13i1e58185_app1.docx]

**Search strategy**

| ***PCC*** | ***#*** | ***Search string*** | ***Hits (Medline via PubMed, 03.03.23)*** |
| --- | --- | --- | --- |
| ***Participation/participatory design/Co-Creation/Co-Design*** | | | |
|  | 1 | participat* [all] | 664,186 |
|  | 2 | co-creat*[all] | 1,540 |
|  | 3 | cocreat*[all] | 1,610 |
|  | 4 | co-design*[all] | 1,706 |
|  | 5 | codesign*[all] | 1,939 |
|  | 6 | co-research*[all] | 408 |
|  | 7 | “patient involve*”[all] | 3,159 |
|  | 8 | “public involve*” [all] | 1,698 |
|  | 9 | “design research*”[all] | 1,592 |
|  | 10 | “designs research*”[all] | 21 |
|  | 11 | “research through design”[all] | 11 |
|  | 12 | co-operat*[all] | 12,813 |
|  | 13 | cooperat*[all] | 262,736 |
|  | 14 | collaborat*[all] | 259,752 |
|  | 15 | contribut*[all] | 1,442,134 |
|  | 16 | user-led[all] | 136 |
|  | 17 | “participatory design”[all] | 572 |
|  | 18 | user-centered[all] | 1,342 |
|  | 19 | user-centred[all] | 458 |
|  | 20 | user-oriented [all] | 231 |
|  | 21 | user-orientated [all] | 12 |
|  | 22 | “community-based participat*” [all] | 7,223 |
|  | 23 | “community-based research*” | 1,388 |
|  | 24 | “human-centered design”[all] | 436 |
|  | 25 | “human-centred design”[all] | 94 |
|  | 26 | “Stakeholder Participation”[MeSH Terms] | 3,865 |
|  | 27 | “Social Participation”[MeSH Terms] | 3,362 |
|  | 28 | “Community Participation”[MeSH Terms] | 47,551 |
|  | 29 | “Community-Based Participatory Research”[MeSH Terms] | 5,911 |
|  | 30 | “User-centered Design”[MeSH Terms] | 180 |
|  | 31 | “Social Inclusion”[MeSH Terms] | 153 |
|  | 32 | OR/#1-#31 | 2,467,281 |
| ***clinical staff*** | | | |
|  | 33 | nurse*[all] | 424,119 |
|  | 34 | “medical doctor*”[all] | 5,551 |
|  | 35 | “medical staff”[all] | 38,808 |
|  | 36 | “medical person*”[all] | 6,905 |
|  | 37 | “health worker*”[all] | 23,133 |
|  | 38 | “health staff”[all] | 2,410 |
|  | 39 | “clinical staff”[all] | 3,569 |
|  | 40 | “nursing staff”[all] | 78,235 |
|  | 41 | “care staff”[all] | 4,406 |
|  | 42 | “hospital staff”[all] | 5,671 |
|  | 43 | nursing[all] | 797,089 |
|  | 44 | therapist*[all] | 42,117 |
|  | 45 | “practical nurse*”[all] | 1,196 |
|  | 46 | “nurse practitioner*”[all] | 26,190 |
|  | 47 | “care worker*”[all] | 17,457 |
|  | 48 | physician*[all] | 682,016 |
|  | 49 | “general practitioner*”[all] | 56,069 |
|  | 50 | doctor*[all] | 185,687 |
|  | 51 | “clinical practitioner*”[all] | 1,043 |
|  | 52 | “health care provider*”[all] | 37,059 |
|  | 53 | “[Nursing Staff](https://meshb.nlm.nih.gov/record/ui?ui=D009740)”[MeSH Terms] | 69,641 |
|  | 54 | [Nurses](https://meshb.nlm.nih.gov/record/ui?ui=D009726)[MeSH Terms] | 97,451 |
|  | 55 | “[Medical Staff](https://meshb.nlm.nih.gov/record/ui?ui=D008503)”[MeSH Terms] | 28,786 |
|  | 56 | [Personnel, Hospital](https://meshb.nlm.nih.gov/record/ui?ui=D010564)[MeSH Terms] | 95,043 |
|  | 57 | [Physicians](https://meshb.nlm.nih.gov/record/ui?ui=D010820)[MeSH Terms] | 173,657 |
|  | *58* | *OR/#33-#57* | *1,852,022* |
| ***AI*** | | | |
|  | 59 | “artificial intelligence*”[all] | 51,367 |
|  | 60 | “artificial intelligence algorithm*”[all] | 557 |
|  | 61 | “machine learning”[all] | 60,369 |
|  | 62 | “machine learning system*”[all] | 307 |
|  | 63 | “explainable artificial intelligence*” [all] | 258 |
|  | 64 | “deep neural network*” [all] | 4,869 |
|  | 65 | “deep learning”[all] | 28,165 |
|  | 66 | “artificial neural network*” [all] | 11,917 |
|  | 67 | “reinforcement learning”[all] | 3,632 |
|  | 68 | “Human-Computer Interaction” [all] | 1,330 |
|  | 69 | “Artificial Intelligence”[MeSH Terms] | 166,652 |
|  | 70 | OR/#59-#69 | 201,559 |
| ***CDSS*** | | | |
|  | 71 | “clinical decision support system*”[all] | 10,207 |
|  | 72 | “clinical decision support program”[all] | 9 |
|  | 73 | “decision support system*”[all] | 13,965 |
|  | 74 | “decision support tool*”[all] | 3,270 |
|  | 75 | “clinical decision support”[all] | 12,029 |
|  | 76 | “medical decision support” [all] | 345 |
|  | 77 | “medical decision support system*” [all] | 175 |
|  | 78 | [Decision Support Systems, Clinical](https://meshb.nlm.nih.gov/record/ui?ui=D020000)[MeSH Terms] | 9,338 |
|  | 79 | “decision support techniques”[MeSH Terms] | 81,985 |
|  | 80 | OR/#71-#79 | 97,702 |
|  | 81 | #32 AND # 58 AND # 70 AND #80 | 169 |
| **+Filters** |  | #81 + Filters: English, German, Abstract, from 2012-2023 | 115 |
