# Supplementary material for: Challenges and Facilitation Approaches for the Participatory Design of AI-Based Clinical Decision Support Systems: Protocol for a Scoping Review
Source: JMIR Res Protoc. 2024 Sep 5;13:e58185. doi: 10.2196/58185 (PMC11413541; doi:10.2196/58185)
Supplement: Multimedia Appendix 2 [file resprot_v13i1e58185_app2.docx]

**Data extraction form**

| ***Item*** |  |
| --- | --- |
| *Author(s)* |  |
| *Year of publication* |  |
| *Country* |  |
| *Aims/purpose* |  |
| *Setting and context (if applicable, eg, hospital)* |  |
| *Type of artificial intelligence (AI) System* |  |
| *Target groups / population (eg, nurses, doctors)* |  |
| *Sample size* |  |
| *Sociodemographic characteristic (eg, age, sex)* |  |
| *Recruitment strategies* |  |
| *Study type / publication type* |  |
| *Definition of participation, co-creation, AI, Clinical decision support (CDSS)* |  |
| *Generic term for the participatory/cocreative process* |  |
| *Participating groups (eg, researchers, informaticians, physicians, clinical staff)* |  |
| *Underlying theories, frameworks, models, theoretical concepts, etc.* |  |
| *Underlying methodologies* |  |
| *Degree of participation/Grading* |  |
| *Described participatory/co-creative approaches* |  |
| *Described participatory and co-creative methods (eg, observational, verbal, written, visual, active methods)* |  |
| *Experience of using a participatory approach or methods* |  |
| *Challenges/limitations, conditions for success and barriers to the participation of clinical staff (mentioned by the author(s) of the original study and/or observed by researchers)* |  |
| *Supporting CDSS development through participatory methods* |  |
| *Aspects of data quality* |  |
| *Aspects of interpretability and explainability* |  |
| *Considering different perspectives on the clinical problem when designing the CDSS* |  |
| *Considered ethical, legal and sociocultural implications (ELSI)/recommendations for ELSI consideration* |  |
| *Identified ELSI* |  |
| *Aspects of fairness, bias and (non-)discrimination* |  |
| *Accountability/responsibility aspects of clinical decision support systems* |  |
| *Quality criteria/assessment of participatory processes* |  |
| *Phases of participation/co-creation (includes among others reflection processes, processes of technology development, piloting and evaluation)* |  |
| *Reported outcomes (eg, Capacity building, learning or emergent knowledge)* |  |
